# Advent of a Link between Ayurveda and Modern Health Science: The Proceedings of the First International Congress on Ayurveda, “Ayurveda: The Meaning of Life—Awareness, Environment, and Health” March 21-22, 2009, Milan, Italy

**DOI:** 10.1155/2011/929083

**Published:** 2010-10-17

**Authors:** Antonio Morandi, Carmen Tosto, Guido Sartori, Paolo Roberti di Sarsina

**Affiliations:** ^1^Ayurvedic Point, C.so Sempione 63, 20149 Milan, Italy; ^2^SSIMA, Italian Scientific Society for Ayurvedic Medicine, C.so Sempione 63, 20149 Milan, Italy; ^3^Atah, Italian Association of Ayurvedic Patients, Via C. Boldrini 14, 40121 Bologna, Italy; ^4^Department of Sociology and Social Research, Observatory and Methods for Health, University of Milano-Bicocca, Piazza dell'Ateneo Nuovo 1, 20126 Milan, Italy

## Abstract

The First International Congress on Ayurveda was held in Milan, Italy in March 2009 and it has been the first scientific event of this kind in western world. This groundbreaking international congress was devoted to human being as the product of interactions between Awareness, Environment and Health, subjects that the West tends to consider separate and independent, but that are believed deeply connected in Ayurveda, whose interdependence defines “The Meaning of Life”. The Congress established a bridge between indian and western philosophy, scientific and biomedical thinking in order to expand knowledge and healthcare. Main attention and address of the invited speakers was on the concept of “relationships” that, connecting living beings with environment, shape Nature itself. This concept is central in Ayurveda but is also common to other western scientific disciplines such as quantum physics and epigenetics that, in the four Sessions of the Congress, were represented by eminent experts. The importance of this event was underlined by the attendance of more than 400 participants and by noteworthy institutional endorsements, that added a significative political dimension of high social impact due to the topical period for CAM acceptance and integration in Europe.

## 1. Introduction

The First International Congress on Ayurveda, “Ayurveda: The Meaning of Life,” organized by the Italian Scientific Society of Ayurvedic Medicine (SSIMA—Società Scientifica Italiana di Medicina Ayurvedica) and the Ayurvedic Point, School of Ayurveda, Milan, Italy [1] and supported by Asthavaidyan Thrissur Thaikat Mooss SNA Oushadhasala, India, was held in Milan, Italy on March 21-22, 2009. The aim of the Congress was to establish a bridge between Ayurveda and Western scientific and biomedical thinking in order to expand the knowledge of health, healthcare, and quality of life [2, 3]. Therefore, the main attention of the featured experts from the fields of Ayurveda and modern science was on the concept of relationships that are central to Ayurveda and the Western scientific disciplines such as quantum physics, epigenetics, and modern medicine. In Ayurveda, the concepts of awareness, environment, and health are intricately linked, and their interdependence defines “The Meaning of Life.” The congress was organized in four Sessions: Shape of Reality, Shape of Environment, Shape of Nutrition, and Shape of Health. 

Professor B. D. Josephson, 1973 Nobel Laureate in Physics (Department of Physics, University of Cambridge, UK), in his inaugural lecture on “Eastern Philosophy and Western Science,” considered the problems with “objective reality” that is central to quantum theory, since reality is too complex to be reduced to a formula in general. There are also a number of well-attested phenomena that seem difficult to accommodate within conventional approaches, and there are also suggestions, originating in the work of Niels Bohr, that biosystems demand the use of “complementary” descriptions [4]. This implies that all descriptions involve a point of view; however, the subjects also should play an important part. Professor Josephson proposed a many-pronged approach that merges the understanding of complex systems, semiotics, and evolutionary mechanisms to achieve a synthesis between the tendency of Western Science to reduce objective reality to a formula, Quantum Mechanics and Eastern philosophy's emphasis on conscious experience with particular reference to Ayurveda.

## 2. The Shape of Reality: Fundamentals of Relations and Interactions

The session discussed the basic principles that intrinsically determine relations and interactions in order to define the shapes of perception. This session was chaired by Professor Ram Harsh Singh (Emeritus Professor, Ayurveda Faculty, Banaras Hindu University, Varanasi, India) whose master lecture on “The Fundamental and Applied Considerations in Srotovijnana of Ayurveda” pointed out the basic concept of Srotovijnana (knowledge of Srotas—channels of relations) as the principal matrix of Ayurvedic biology and medicine [5]. The word Srotas is derived from the Sanskrit root “Sru- Gatau” which has a wider meaning that includes, but not limited to, going, moving, continuing, connecting, filtering, flowing, leaking, and secreting. The Ayurvedic classics proclaim “Srotomayam hi Shariram” meaning that the living physical body is a channel system and/or is comprised of innumerable channels designed as an inner transport system for divergent functions, both gross and subtle, tangible and intangible, biologic and energetic. The entire range of life processes in health and disease depends on the integrity of the Srotas System which is prone to lose its integrity due to out-of-order lifestyle, faulty food, and day-to-day wear and tear warranting periodic Samshodan or biopurification for which Ayurveda has developed its therapeutic technology popularly called “Panchakarma” therapy. He affirmed that Ayurveda can be better understood in terms of philosophy and physics than by modern reductionist biology [6]. Ultimately, the study of the full spectrum of the Srotas and function will help to define the phenomenon of relationships in structural and functional biology [7]. 

Professor Alex Hankey (Professor, Department of Yoga and Physical Sciences, SVYASA, Bangalore, India), in “The Ayurvedic Concept of Perfect Health,” presented how the Ayurvedic concepts of underlying health restoration and maintenance could be understood through a modern understanding of quantum theory [8]. He emphasized that it is possible to achieve and maintain a state of “perfect health” by gaining insight into the nature of reality, commonly associated with enlightenment. In this, the sense perception is seen as a “manifestation,” and not “objectively real,” as validated by the recent “manifest universe” interpretation of quantum theory. A new approach, uniting thermodynamics and quantum theory, states that a lack of thermodynamic equilibrium is the basis for physical manifestation, analogous to the lack of equilibrium as defined in Vedic sciences with regard to “gunas,” the inherent qualities of differentiated matter—pure quiescence, dynamic motion, and state of inertia. 

Swami K. K. S. Joythimayananda (Ashram Joytinat, Italy) described his 50-year experience in Ayurveda and Yoga from both spiritual and materialistic perspectives. The purpose of Yoga and Ayurveda is health, happiness, and liberation in order to acquire profound knowledge. Yoga and Ayurveda simply offer directions to physical activity, nutrition, and rest. Ego, ignorance, greed, and attachment are great pollutants because the idea of possession of objects creates insufficiency or stress. The act and awareness of being is the reality in which truth is embodied and silence is enjoyed. By contrast, the wrong perception, the Maya or illusion that often perceives stress or insufficiency, directs the individual away from the truth. A correct perception transcends all, while a wrong one generates suffering and illness.

Dr. Rama Jayasundar (Department of Nuclear Magnetic Resonance, All India Institute of Medical Sciences, New Delhi, India), in her presentation on “Quantum Logic in Ayurveda,” explained how quantum physics has revealed some concepts similar to those discussed in Vedas and in Ayurveda as well (Ayurveda being rooted in the Vedas) [9]. Ayurveda understands the human body as interconnected within as well as outside. By integrating the role of mind and consciousness in the human body, Ayurveda is distinctly different from biomedicine's Newtonian physics-based perception of the organism as a structural entity made up of fundamental units of building blocks, that is, atoms and molecules [10]. Even, while accepting the reality of the physical body, Ayurveda emphasizes the role of consciousness and mind in both health and disease states. This is in contrast to biomedicine, which relates physiology to structures. Dr. Jayasundar emphasized the concept in Ayurveda that one's thoughts create health or illness which is similar to the quantum physics' concept that thoughts create the physical reality [11].

Dr. Gianluigi Marini (Director, Oncology Department, Saint Anna Hospital, Lugano, Switzerland) presented a novel interpretation of the dualism of health and disease with “A New Paradigm For Cancer: From the Extreme Expression of Organic Disease to the Outstanding Example of Functional Pathogenesis of Disease.” He suggested that health and disease are forms of reality derived from structured information interwoven among source points. These sources, defined by the information transported by channels (Srotas), are the necessary conditions to maintain existence and health. A block in this flow of information results in disaggregation of the system at all levels of existence: molecular, cellular, tissual, organic, psychological, interactional, and social. The extreme disaggregation is cancer, a condition where all levels of relationship become deranged. The prerequisite to reestablishing health is acknowledging the sources of reality, restructuring the channels, and resetting the proper inflow and outflow.

## 3. The Shape of Environment: Recursive Adaptive Responses

The session aimed at describing how continuous adaptive feedback between human beings and environment recursively links and shapes these two entities, defining the functional and dynamic unity between observers and the observed. The final outcome is health or disease. Chairing the session, Dr. Darshan Shankar (Chairman, Indian Ayurvedic and Integrative Medicine Institute, Bangalore, India), in his presentation on “The Challenges of Integrating Traditional Knowledge and Science”, focused on the epistemology of traditional Indian knowledge and science using the theoretical foundation of Ayurveda. Many modern scientists believe that traditional knowledge is irrelevant to contemporary needs, a view that originated in colonial and postcolonial history when a political strategy of domination diminished the value of “indigenous” knowledge systems. The ways of knowing about nature and environment in traditional knowledge systems and science are fundamentally different, as are the depth, range, and scope of knowledge they discover. Modern science has a detailed knowledge about parts of physical and biological nature, whereas traditional knowledge systems have a holistic knowledge of the physical, biological, and spiritual fields that pervade nature. One of the greatest challenges of this century will be integrating the reductionist framework of science with the holistic framework of traditional knowledge systems in order to see the whole in the part and the parts in the whole.

Dr. Mark Rosenberg (Director, European Academy of Ayurveda, Birstein, Germany), in “Vastu Shastra—The Vedic Concept of Holistic Architecture for Healthy Living,” analyzed the logic of Vastu Shastra, the Vedic knowledge for construction of buildings. Vastu is based on the observation that certain spatial and environmental conditions, combined in harmony with the elements, were determinants in the construction of temples, houses, and buildings. Such structures were respectful, of natural proportion, promoting health and prosperity of the inhabitants since the proportions of nature connect the human being with it. Another basic topic of Vastu is the influence of magnetism on the spatial objects and the building materials that were used. Dr. Rosenberg highlighted how magnetic therapy is able to give effective healing, and magnetic waves, which permanently flow from North to South, also play an important role for wildlife. The existence of a well-regulated magnetism sustains a positive energy field within the ambience, supporting the preservation of physical health and mental vitality.

Dr. Ernesto Iannaccone (Teacher of Sanskrit and Ayurveda Classical Texts at “Ayurvedic Point” School of Ayurvedic Medicine, Milan, Italy), in “Ecological Awareness in Ayurvedic Ancient Texts,” pointed out that in all the classical Vedic and Ayurvedic literature, a unifying factor aims to bring the shapes and events of nature back into an essential oneness, where differences dissolve. Ancient Indian thinkers believed in a deep interrelation between living beings and the expressions of nature. Human beings are inseparable from nature; therefore, Ayurvedic ethics stress respectful behavior towards the environment. Ayurvedic authors were well aware of the risks of environmental degradation; the third chapter of Vimanastana of Charaka Samhita, one of the most ancient treatises on Ayurveda, elaborates extensively on the causes and consequences of the deterioration of climate, water, and land. 

Professor Francesco Donato (Head, Institute of Hygiene, Epidemiology and Public Health University of Brescia, Italy), in “Ayurveda and Modern Western Medicine: A Scientific Approach Toward a Possible Integration,” presented the difficulties of adapting the objectivity of Western clinical trials to the Ayurvedic concept of interaction and interrelation between man and environment. Since the 1990s, the increasing popularity of Ayurveda in the West has spawned interest in scientific evaluation. There has been limited modern evidence-based investigation of the holistic approach of Ayurveda to the health of an individual, its diagnostic and therapeutic methods, or its disease prevention and health promotion recommendations. Since the researches are still in an evolving phase with regard to approaches and methodologies, the trends are not easily evaluable at this stage. Only recently pharmacogenomics and biostatistical approaches have been used to investigate the scientific basis of the three “Dosha” theories [12]. However, more research in this field has a potential and should be encouraged with scientifically sound studies including well-designed clinical trials assessing the multidimensional approach of Ayurveda.

Dr. Madan Thangavelu (Research Fellow, MRC—Cancer Cell Unit and Department of Oncology, University of Cambridge, UK), in “Human Genomes, Genomic Variation and Environmental Modulators of Health and Disease,” reviewed the changing perspectives of the human genome—variation between personal or individual genomes, the rich concepts of epigenomics and human microbial metagenomics, transgenerational nongenomic mechanisms, and the developmental origins of health and disease. All such new knowledge pose major challenges for reconciling promises of genomic appreciation of health and disease. Using the “Average Human Genome of 2005” as a baseline, Dr. Thangavelu reviewed evidence for levels of genomic variation among individuals, the difficulty in mapping genetic changes over time as in natural aging, the genomic changes in disease, and the interplay between genes/genomes and the environment in diverse states of health and disease. These revolutionary concepts in contemporary biology, he reminded, echoed ancient knowledge. In such a turbulent milieu, the distinction between the environment and the genetic modulators of health and disease was further blurred. Microenvironments ranging from intracellular, intercellular, tissue, organs, systems, to intersystem levels—similar to the Ayurvedic concept of “srotas”—had to be taken into account for appreciating the scale of such influences. Using examples of mucin accumulation in pancreatic ductal epithelial cells, he argued how contemporary cell biology of pathogenesis could benefit greatly by appreciating the ancient concepts of “Srotas,” “srotodusti,” “agni,” “ama” mala, and others. Such Ayurvedic descriptors can enable the correlation of subtle changes in organ, tissue, and cell and molecular biology parameters to the “shatkriyakal” process—the six stages of progress towards disease—described first in Sushruta Samhita. The adaptation to environmental changes that the human body accommodates to maintain good health and mediated by cellular processes could well be seen through and studied by using such descriptors. The continuing conflict between Science and the Knowledge of Life, he reminded, was also not a surprise for the Ancients.

## 4. The Shape of Nutrition: Nutrition for Transformation of Body, Mind, and Behavior

The session focused on the “matter/information” transported by “Srotas”/“Relationships” which defines various stations of the relations linking largely with the concept of “food.” Vaidya Atreya Smith (Director, European Institute of Vedic Studies, Sauve, France), in “Nourishment According to Ayurveda,” showed that “food” in Ayurveda has a very broad meaning which is transformed from the heterologous into the homologous. Charaka Samhita, one of the oldest texts in Ayurveda, states that nourishment is derived from “Rasa,” a Sanskrit word whose root-meaning carries numerous connotations such as juice, liquid, fluid, or essence of any plant or living being and taste as a sense, love, affection, and desire. In Ayurveda, it has to be understood in context. Nourishment should invoke a profound physical and psychological effect, and as such, even a substance containing all necessary vitamins and minerals may still not be considered “nourishing.” Metaphorically, the scent of a favorite childhood meal can stir powerful memories of love and affection, indicating that something more than tissues and fluids are being nourished by food. Thus, from the Ayurvedic point of view, our senses interact with food to extract nourishment, pleasure, happiness, and even love.

Speaking on “Ayurveda and Modern Science,” Dr. Nikolaos Kostopoulos (Director, Holistic Health Center, Athens, Greece) offered insights on how Ayurveda has acquired an important role in the understanding and management of disease in the modern world. Western medicine is extremely efficient in acute conditions that need potent medications, hospital care, or high technological support. However, the leading causes of mortality nowadays are related to incorrect lifestyle, poor nutrition, and stress. Modern medicine treats mostly external factors or intervenes in the biochemical chain of events, that is, by eliminating bacteria, substituting hormones, or supplementing nutritional deficiencies. In contrast, Ayurveda analyzes the person apart from the disease by classifying people into different constitutions with distinct characteristics and susceptibilities that determine the type of treatment required. The cornerstone of Ayurveda is what modern science tries to analyze via genetics. Moreover, Ayurveda places great emphasis on mind and the individual psychological state in prevention and treatment of disease. In Ayurveda, the health of the individual, the society, and the environment are intricately connected.

Dr. Ashtavaidyan A. N. Narayanan Nambi (Director, Research and Education Division, Ayurveda Institute SNA Oushadhasala, Thrissur, India), in his narration, “The Nature of Nutrition and the Nutrition of Nature,” suggested that often a human being distinguishes the qualities of food, but being not well supported by the inner self, he/she becomes a slave to the senses. Hence, the realization of the self within the context of time and space will result in the appropriate selection of that food which nourishes best. What we eat will decide how we think, and what we create is what we think.

Professor Francesco Marotta (Department of Gastroenterology, Saint Giuseppe Hospital, Milan, Italy and Scientific Director of GAIA Foundation), in “Human Nutrition in Genomic Medicine: An Inside-Out Story,” explained that Ayurveda considers aging as Swabhava, or the nature of a time-bound living being that ceases to exist biologically through senescence and death, though its energetic body survives. Even so, Ayurveda also points out that appropriate life-style, nutrition, and rejuvenative Rasayana remedies can reverse the progressive loss of biological qualities associated with specific phases of life. Interestingly, there are some conceptual similarities in current scientific achievements. Nutrigenomics studies the genetic and epigenetic interactions with a nutrient that leads to a phenotype change. This entails the understanding of how nutrition affects metabolic pathways, influences diet-related diseases, and plays a role in individual genotypes. This new scenario requires multifaceted team interplay and cannot rely on a simplistic, single-target drug approach.

Dr. Guido Sartori (Vice President, Italian Scientific Society for Ayurvedic Medicine, Italy), in “Plants, Biovitality and Ecocompatibility,” described the Ayurvedic view that plants are a source of nutrition and medicines. A complex and refined Ayurvedic pharmacology aims to make the inner properties of plants safely bioavailable. The peculiar characteristic of vegetal organisms is their vitality which is based on the same constitutive elements as human beings, validating the use of plants in Ayurveda. Plants live and grow in an environment that has to be suitable for them, free from contaminants and pollutants. The maintenance of the soil, water, and seeds requires the care and attention that only people who are aware and respectful of the ecosystem can give. Ayurveda gives criteria to make ecocompatible, diverse choices that are linked to cultivation for the benefit of the health of all living beings.

## 5. The Shape of Health: Interactions and Interrelations as Intrinsic Determinants of the Status of Health

The last session focused attention on health as the harmonic outcome of a proper balance among the systems of mutual relationships determining the reality of living beings. Dr. G. G. Gangadharan (Joint Director, Foundation for Revitalization of Local Health Traditions, Bangalore, India), who chaired this session, in his exploration on “How the Idea of Relationships/Srotas Can Determine the Concept of Diagnosis in Ayurveda,” talked about the gross channels particularly their clinical and pathological aspects reconnecting with Professor Singh's emphasis on the basis concepts of Srotovijnan of Ayurveda. Physicians generally consider the Srotas (channels), which have physical expressions, as nutrients/biological fluid transporters. The body is free from disease when the Srotas functions normally. If the Srotas gets occluded or malfunctioned due to internal or external factors, the altered flow creates an accumulation of products, mainly toxic, leading to functional, and later, organic alterations. More deeply, the concept of Manovaha Srotas (mind-body channels) brings together the structural and nonstructural or physical and nonphysical Srotas. Thus, Ayurvedic physicians describe pathogenesis in terms of structural or functional alteration of the Srotas of body and mind. Dr. Gangadharan concluded that the analysis of Srotas plays a very important role in the diagnosis of disease. 

The importance of Ayurvedic evaluations of constitutional types and their balance as health indicators was highlighted by Professor Bhushan Patwardhan (Director, Interdisciplinary School of Health Sciences, University of Pune, India). In “Ayugenomics: Ancient Concepts—Modern Pharmacogenomics,” he reported on evidence reported by his laboratory. Ayurveda classifies humans into three major constitutional types (Prakriti) based on distinct morphological, metabolical, and psychological characteristics called Prakriti: Vata, Pitta, and Kapha. These types may offer phenotypic datasets suitable for analysis of underlying genetic variation. The prognosis, diagnosis, and therapeutics in Ayurveda are Prakriti specific and have similarities with modern concepts of pharmacogenomics. Ayurveda can provide the data sets for phenotypic classification irrespective of ethnicity, geography, and race. As proof of this concept, his study evaluated 76 subjects both for their Prakriti and HLA DRB1 types and found some significant associations [13]. New study is underway to investigate the genetic basis of Prakriti as a major prognostic, diagnostic, and therapeutic tool through genomic variation analysis and gene expression profiling of human Dosha Prakriti. Such an effort could provide good leads and may form the basis of future customized medicines.

Health is the result of a balanced network of interactions and interrelations within living beings and their environment. In “Relationships of the Immune System and its Alteration during Cancer,” Dr. Senthamil Selvan (Principal Scientist/Associate Scientific Director, Department of Cell Biology, Hoag Hospital Cancer Center, Newport Beach, California, USA) critically analyzed the relationship between cancer and immune cells. Cancer is a disease of uncontrollable cell division due to loss of normal regulatory controls in the cells as a result of genomic mutations and/or epigenetic mechanisms. Dr. Selvan illustrated how the cross-talk between the immune system and cancer ultimately shapes cancer elimination, evasion, or even progression. He referred to his cutting-edge combinatorial personalized cancer treatment clinical trial with tumor-reduction surgery followed by vaccination with tumor-associated antigens that is aimed at boosting the immune system to ultimately eliminate tumors [14, 15]. He evaluated that various immunotherapeutic clinical trials used so far have only yielded benefit to a subset of patients [16]. Such results are due to evasion of host immune attack induced by the ever-growing complex network of tumor-dependent mechanisms and immune system-dependent mechanisms. As a consequence, tumor-induced immunological alterations and, in turn, immune system-induced tumor alterations are a component of the complex tumor-immune relationship. He envisioned that this interaction, advantageously, opens up avenues to better understand the dynamics of the Ayurvedic medical approaches including Rasayanas, which is to curtail growth of cancer cell and to stimulate the immune system, and also to combine the ancient comprehensive approach with modern medicine to strengthen immunological reactivity against cancer. 

Dr. Ram Manohar (Research Director, Ayurvedic Trust (AVT) Institute for Advanced Research, Coimbatore, India), in his analysis on “Research in Ayurveda: Challenges and Solutions,” determined research as an integral component of developmental activities in Ayurveda today. Though globalization is driving the demand for evidence of safety, efficacy, and quality of Ayurvedic medicines and treatments, an approach to clinical research is needed that should not compromise Ayurveda's holistic approach. To tap the full potential of Ayurveda, holism must be preserved in both practice and research. Dr. Manohar shared the striking results of a 36-week, controlled, randomized double-blind clinical trial to test the efficacy of Ayurvedic medicines versus Methotrexate (MTX), their combination, and placebo in Rheumatoid Arthritis [17]. The study, financed by a PICRC Grant of NCCAM NIH, USA, was conducted in India in collaboration with the University of  Washington, Seattle, the University of California, Los Angeles, and The Ayurvedic Trust, Coimbatore, India. Preliminary findings indicate that MTX and Ayurvedic treatments were approximately equivalent, and their combination did not improve efficacy. There were fewer adverse effects in the Ayurveda group. The trial was characterized by individualization of Ayurvedic treatment, change of medications during study visits, and introduction of placebos for classical Ayurvedic medicines. 

Finally, as a practical demonstration of the importance of the social environment in health care and prevention, Dr. Carmelo Scarcella (General Director, ASL - Local Health Authority of Brescia Province, Italy) presented “Complementary Medicine Integrated with Modern Western Medicine: An Experience in a Local Health Authority in Italy.” The Brescia ASL is one of the largest in Italy covering one million inhabitants, including 12.5% registered immigrants, in a highly industrialized area. In response to consumer demand, the Brescia ASL has promoted the use of Complementary and Alternative Medicine (CAM), integrated with modern Western medicine, since 2001. In 2005, the ASL formed a nonprofit association, the Italian Centre for the Study of Oriental Medicine (CISMO), which organized introductory courses on Ayurvedic medicine for doctors and pharmacists and participated in scientific meetings on CAM and migration medicine in Italy and in the International Arogya Conference, held in Delhi in 2007. The ASL and CISMO have conducted scientific studies on CAM, including a randomized, controlled trial on Ayurvedic herbs for the treatment of hypercholesterolemia and obesity with the cooperation of University of Brescia. An agreement to cooperate for scientific research on Ayurveda was signed between CISMO, the AYUSH Department, and Ministry of Health and Family Welfare, Government of India, in 2006.

## 6. Education, Research, and Bioethics of Ayurveda in the Western World: Developing Global Regulations

The increasing popularity of Ayurveda in the Western world health care context is creating the need for careful regulation and organization of its education and research. Dr. Giuseppe Tucci, the foremost founder of the Italian Orientalism, in his presentation on “India is the spiritual organ of Asia,” stressed the urgency of awareness to protect Indian Traditional Medicine for its wisdom as Human Heritage. Ayurveda, first of all, and Anthropological/Traditional Medical Systems emphasize a person-centered, humanized approach to medicine based on people's whole bio-psycho-spiritual unity and equilibrium, including their relation with the environment and the way they perceive or “narrate” their own complex individual existence in sickness and in health [18, 19]. The World Health Organization has adapted the term “Traditional Medicine” and urges the necessary ethics to protect, safeguard, promote, study, and apply the cultural heritage of Western and Eastern medical and anthropologic health knowledge and systems. There is a growing demand among the Western world for a traditional-mind-shape approach to the multidimensional needs of the triad of disease-illness-sickness. In many cases, clinical effectiveness of certain Ayurvedic and Traditional medications has been found comparable to the efficacy of conventional Western medicines [20]. The Traditional Medical Systems are also very safe and, therefore, can help to reduce the enormous burden of mortality and morbidity caused by the adverse effects as well as the iatrogenic effects of conventional prescription drugs, ever-increasing resistance to antibiotics, and the inability of biomedical drugs and therapies to cope with and manage chronic and psychosomatic diseases [20]. Current attitudes to health include a preference for natural products over chemical drugs, a holistic sustainable philosophy and related behavior in terms of health management, a belief in individual responsibility for achieving wellness, and more attention for emotional needs. 

Ayurveda and other Traditional Medicines and Nonconventional medicines can often be used as a first option to treat certain problems, keeping more costly, more invasive, and potentially toxic treatments as a second option [20]. This was emphasized in the presentation by Dr. Ram Manohar from The Arya Vaidya Pharmacy (AVP), Coimbatore, India with particular reference to an Ayurvedic treatment approach for rheumatoid arthritis. The Department of AYUSH (Ayurveda, Yoga, Unani, Siddha, and Homeopathy), Ministry of Health and Family Welfare of the Indian Government, plans to establish collaboration with various countries across the world in the fields of education, health care, and research in Ayurveda. Ayurveda training, research, and practice in healthcare need to be adapted in Europe and other Western world countries to a different social, political, and educational environment in order to allow the integration of Ayurvedic Medicine in current medical practice and diverse Health Care Systems without compromising the principles of Ayurveda [20, 21]. The discussion by the working group highlighted the paramount importance of creating synergy among the efforts of the Department of AYUSH, India and the various institutions, schools, and Nongovernmental Organizations (NGOs) working outside India for propagation of Ayurveda. Recently, the Department of AYUSH received requests from several NGOs based in Europe and the USA (which are merging in the International Alliance of Ayurvedic Professional Associations—IAAPA) to open a dialogue so that a cooperative mechanism can be worked out to ensure quality and professionalism in training, research, and practice of Ayurveda. In Italy, Ayurvedic and the other Nonconventional Medicines already have been recognized through a medical act (since 2002) by the Italian National Federation of Councils of MDs and Dentists (FNOMCeO). The Italian Scientific Society of Ayurvedic Medicine SSIMA, “Ayurvedic Point,” and the Italian Association of Ayurvedic Patients “Atah” are all founding members of the Permanent Committee of Consensus and Coordination for Nonconventional Medicines in Italy and will continue to emphasize the need to develop acceptable global regulations to bring traditional medicines into the mainstream [22].

In summary, the first ever international congress on Ayurveda in the West established the ground work for building a bridge between Indian and Western philosophy of scientific and biomedical thinking in order to improve healthcare. The main focus of the speakers was on the concept of relationships that connect living beings with the environment, which is to shape Nature itself. This concept is the centerpiece in Ayurveda but is also common to other Western scientific disciplines such as quantum physics and epigenetics. The importance of this event was underlined by noteworthy international endorsements that held a significant political-social impact in this contemporary period of CAM acceptance, integration, and creation of international multidisciplinary studies on the relationships between the Ayurveda basic such as (a) “Panchamahabhutas” and modern physics and biology; (b) “Srotas,” “srotodusti,” “agni,” “ama,” “shatkriyakal”, and such other descriptors that are chartable to processes and changes in organ, tissue, cell, and molecular biology parameters; and (c) “Rasayanas” and pharmacoimmunomics.
